# Esthetic Rehabilitation of Maxillary Anterior Teeth, Including an Immediate Provisionalization with an Implant-Supported Fixed Dental Prosthesis

**DOI:** 10.3390/jcm8040428

**Published:** 2019-03-28

**Authors:** Kyung Chul Oh, Jeongwon Paik, Jee-Hwan Kim

**Affiliations:** 1 Department of Prosthodontics, Yonsei University College of Dentistry, Seoul 03722, Korea; kyungabc@yuhs.ac; 2 Department of Periodontics, Yonsei University College of Dentistry, Seoul 03722, Korea; jpaik@yuhs.ac

**Keywords:** dental implants, immediate provisionalization, computer-aided design, soft tissue dimensional changes, reverse engineering software, esthetic rehabilitation

## Abstract

This report describes the case of a patient who required rehabilitation of their maxillary anterior teeth following a traumatic injury through a physical altercation. The decision was made to extract the maxillary central incisors and maxillary right lateral incisor, perform immediate implantation on the maxillary right lateral incisor and left central incisor areas, and place a three-unit immediate provisional restoration. Predesigned virtual teeth enabled efficient fabrication of the immediate provisional restoration following the implant placement. After a sufficient healing period with periodic check-ups, final impressions were made using a digital approach, with meticulous care taken to preserve the gingival architecture around the sites of rehabilitation. Thus, the custom abutments and definitive restoration were placed without eliciting an uncomfortable feeling in the patient. Both esthetic and functional outcomes were satisfactory. Reduced soft tissue volume around the implant restoration was observed, primarily within the two months post-extraction/implantation, based on superimposition of the serial scan data. Soft tissue volume changes in the present case suggest the need for controlled clinical studies of three-dimensional changes of gingival contours after extraction and/or implantation.

## 1. Introduction

The application and development of digital technologies have extended the scope of implant dentistry [1,2]. Radical improvements in diagnostic imaging devices, as well as computer-aided design/computer-aided manufacturing (CAD/CAM) equipment, have enabled dentists to implement prosthetically-driven implant placement [3,4,5,6,7,8,9]. For many years, it has been recommended to perform a diagnostic wax-up procedure at the site of future implant placement, in order to achieve this “top-down” approach [10,11,12,13]. This procedure can now be completed by virtually arranging the teeth in implant-planning or CAD software [14,15]. Subsequent procedures following implant placement, such as making final impressions for implant prostheses, can also be performed by a digital approach with an intraoral scanner and a scan body [3,16].

Immediate placement of a fixed-type provisional crown following single-implant placement is regarded as a treatment option that is predictive of satisfactory outcomes, provided that the case selection is conducted appropriately [17,18,19]. This may benefit patients from an esthetic perspective, because it eliminates the edentulous stage. Moreover, when fabricated with an accurate three-dimensional (3D) position, the provisional crown can help to maintain the height of the interproximal papillae [20,21]. Immediate provisionalization using one-piece implants was suggested as a treatment strategy to restore partially edentulous anterior teeth in the mandible or lateral teeth in the maxilla, [22,23]. However, there have been few reports regarding the immediate placement of a fixed-type provisional restoration after multi-implant placement in the maxillary anterior tooth region [24]. Bone grafting around the gap between the immediately placed implant and the surrounding bone has been suggested to preserve soft tissue contours and buccal plate thickness [25]. With advances in digital technology, reverse engineering software can provide a comprehensive visualization of dimensional changes that may have occurred after extraction.

This report describes the rehabilitation of an anterior tooth region with multiple implants—including an immediate provisionalization with an implant-supported fixed dental prosthesis in a fully digital workflow—and demonstrates three-dimensional changes in gingival tissues around the sites of rehabilitation over a one-year period.

## 2. Case Report

**Figure 1:**
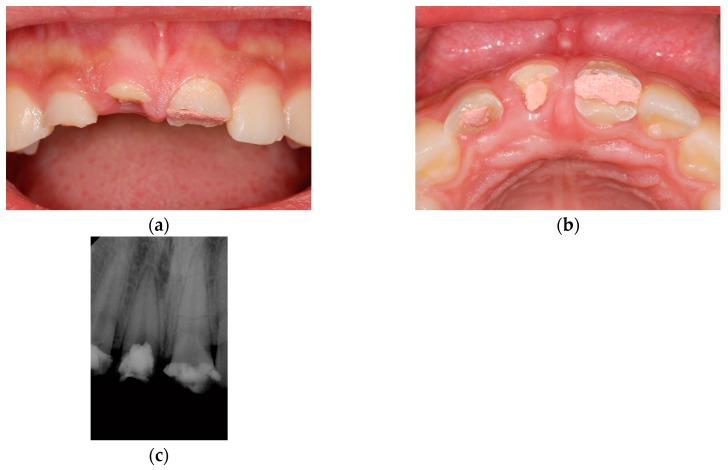
Clinical photographs and a periapical radiograph taken at the initial visit; (**a**) Frontal view; (**b**) Occlusal view; (**c**) Periapical radiograph.

This study was conducted in accordance with the Declaration of Helsinki, and the protocol was approved by the Ethics Committee of Yonsei University Dental Hospital (2-2019-0006). An 18-year-old man requested rehabilitation of teeth that had experienced trauma on the maxillary arch after a physical altercation (Figure 1). Although he had received emergency root canal treatments on the maxillary central incisors and maxillary right lateral incisor within a few days of this event (approximately one month before his visit to our clinic) to relieve pain, these teeth exhibited a poor prognosis. The diagnosis was a Class III crown/root fracture on the maxillary right central and lateral incisors, and a Class III crown fracture with a horizontal root fracture on the maxillary left central incisor. Hence, the decision was made to extract these three teeth, perform immediate implantation on the maxillary right lateral incisor and left central incisor areas, and place a three-unit immediate provisional restoration.

**Figure 2:**
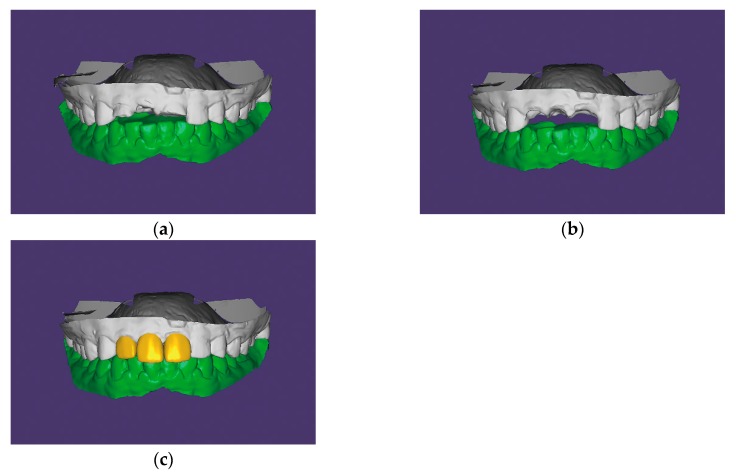
Diagnostic virtual tooth arrangement procedures in CAD software (Exocad DentalCAD version 2015.03, Exocad GmbH, Darmstadt, Germany); (**a**) Maxillary and mandibular diagnostic casts scanned with a tabletop scanner (Identica Blue, Medit Co., Seoul, Korea); (**b**) Modification of the sites planned for rehabilitation; (**c**) Design of the virtual teeth on the modified maxillary diagnostic cast. CAD, computer-aided design.

The maxillary and mandibular diagnostic casts obtained from the preliminary impressions were scanned with a tabletop scanner (Identica Blue, Medit Co., Seoul, Korea) (Figure 2). The teeth that were to be extracted were removed from the maxillary diagnostic cast to arrange the virtual teeth within CAD software (Exocad DentalCAD version 2015.03, Exocad GmbH, Darmstadt, Germany). The virtual teeth were designed on the modified diagnostic cast to ensure that they exhibited no occlusal contacts with antagonists. Together with the remaining teeth on the same arch, the arranged virtual teeth were saved as a unit in a standard tessellation language (STL) file format. A cone-beam computed tomography scan was taken to design and fabricate a surgical guide. The guide was designed in the implant planning software (Implant Studio version 2.17.1.4, 3Shape, Copenhagen, Denmark) and fabricated using a 3D printer (Zenith U, Dentis, Daegu, Korea).

**Figure 3:**
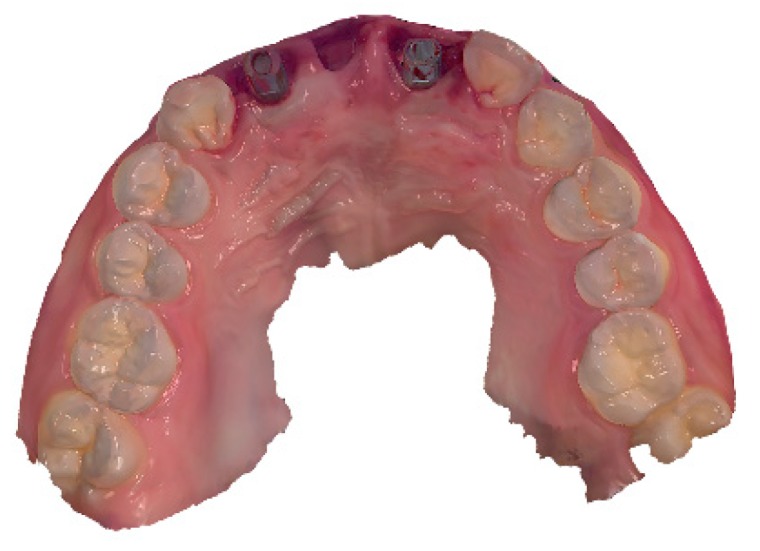
Digital intraoral impressions obtained with an intraoral scanner (Trios 3, 3Shape, Copenhagen, Denmark) and scan bodies (Geo Scanbody, GeoMedi, Uiwang-si, Korea).

Bone-level implants (Straumann BLT (Institut Straumann AG, Basel, Switzerland) 4.1 × 12 mm implant on right lateral incisor area and Straumann BL (Institut Straumann AG) 4.1 × 12 mm implant on left central incisor area) were placed using a flapless approach. Subsequently, the scan bodies (Geo Scanbody, GeoMedi, Uiwang-si, Korea) were connected to the implants and digital intraoral impressions were made using an intraoral scanner (Trios 3, 3Shape, Copenhagen, Denmark) (Figure 3). The gaps between the implants and bony walls were filled with a deproteinized bovine bone mineral (Bio-Oss, Geistlich Pharma AG, Wolhusen, Switzerland). A bioresorbable collagen membrane (Bio-Gide, Geistlich Pharma AG) was then carefully applied to cover the graft material [26]. For the future pontic (right central incisor) area, a ridge preservation technique was performed using a demineralized bovine bone mineral with 10% collagen (Bio-Oss Collagen, Geistlich Pharma AG) without primary soft tissue closure [27,28].

The provisional restoration was prepared during the time the periodontist was performing these supplementary procedures. A new project file was created in the same CAD software and the digital intraoral impressions were imported. The initially predesigned virtual teeth and modified maxillary diagnostic cast assembly were then imported into the software and superimposed over the digital intraoral impressions. The arbitrarily arranged virtual teeth, which appeared on the project file as a default function of the software, fit onto the predesigned virtual teeth to automatically find their predetermined positions by employing a series of software functions (Video S1). Minor adjustments were made to finalize the design of the provisional restoration.

**Figure 4:**
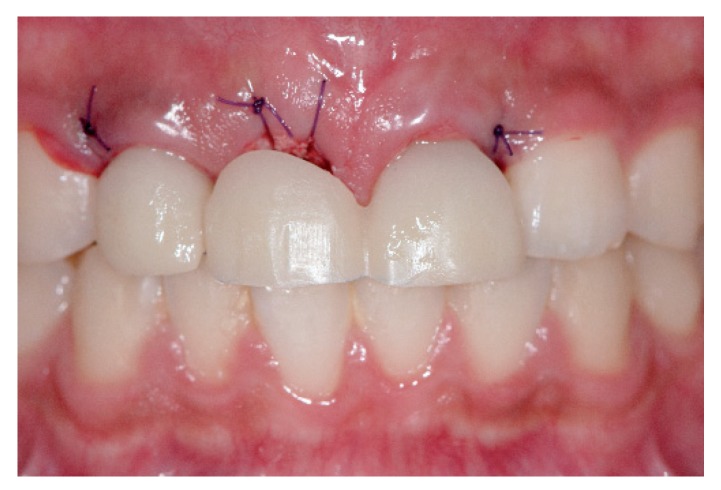
Placement of a set of provisional restorations immediately after implant surgery.

Two sets of provisional restorations from the same design were prepared; they were milled with a poly(methylmethacrylate) resin block (VIPI Block Trilux, Dental VIPI Ltda, São Paulo, Brazil) and bonded to the titanium link abutments (Geo Multibase Abutment, GeoMedi, Uiwang-si, Korea) with a resin cement (Multilink N, Ivoclar Vivadent, Schaan, Liechtenstein). One set of restorations was placed in the patient’s mouth on the day of the implant surgery (Figure 4). This immediate provisional restoration was sufficiently adjusted to provide relief above the ridge preservation area and allow interpapillary growth. Most importantly, occlusion was evaluated and adjusted in order to avoid contacts with antagonists, which inevitably compromised the esthetics to some extent.

**Figure 5:**
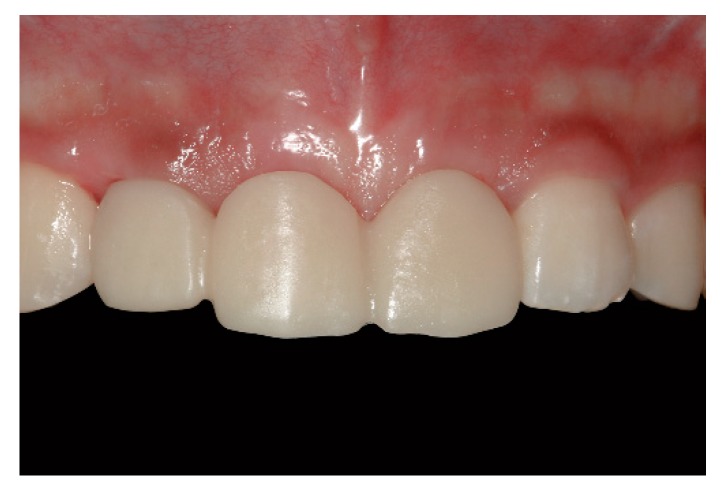
Placement of the other set of provisional restorations at 2 months postoperation.

**Figure 6:**
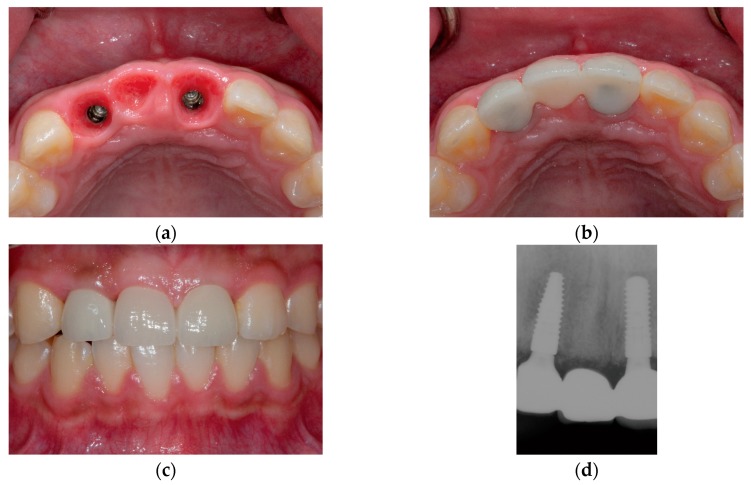
Clinical photographs and a periapical radiograph of the definitive porcelain-veneered zirconia fixed dental prosthesis; (**a**) Occlusal view after removal of the provisional restoration, showing healthy mucosa profiles; (**b**) Occlusal view after placement of the definitive restoration; (**c**) Frontal view after placement of the definitive restoration; (**d**) Periapical radiograph.

The other set of provisional restorations was placed in the patient’s oral cavity 2 months after the initial visit (Figure 5). At this time, minor adjustments were conducted for the restoration, which resulted in better esthetic outcomes. Final impressions were made in the digital approach, with meticulous and specific emphasis on preserving the gingival architecture around the sites of rehabilitation. Custom abutments and the definitive porcelain-veneered zirconia fixed dental prosthesis were fabricated and placed in the patient’s mouth (Figure 6). Both esthetic and functional outcomes were satisfactory.

**Figure 7:**
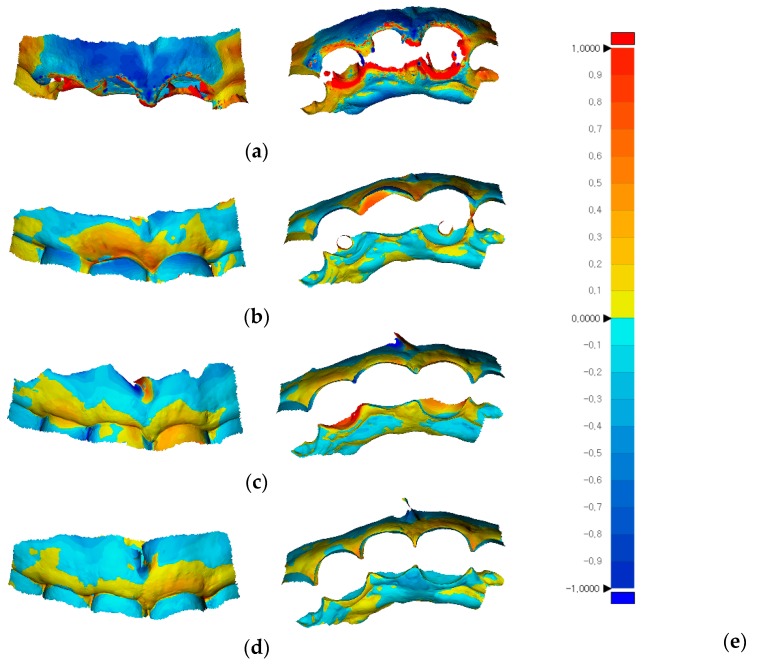
Soft tissue dimensional changes around sites of rehabilitation, shown as color maps using reverse engineering software (Geomagic Control X version 2018.0.1, 3D Systems, Cary, NC, USA); (**a**) Superimposition of the 2-month postoperative state over the state exhibited immediately after extraction (RMS: 757.6 μm); (**b**) Superimposition of the 4-month postoperative state over the 2-month postoperative state (RMS: 221.1 μm); (**c**) Superimposition of the 6-month postoperative state over the 2-month postoperative state (RMS: 286.7 μm); (**d**) Superimposition of the 1-year postoperative state over the 6-month postoperative state (RMS: 128.1 μm). RMS, Root mean square; (**e**) Color-scale bar showing maximum and minimum critical values of ± 1,000 μm.

To record the dimensional changes around the rehabilitated areas, digital intraoral impressions were made five times: immediately after extraction, and at 2, 4, 6, and 12 months after the date on which the extraction and implantation were performed; these corresponded to specific time points, as shown in Table 1. Soft tissue dimensional changes around the sites of rehabilitation were visualized as color maps using the best-fit alignment function in the reverse engineering software (Geomagic Control X version 2018.0.1, 3D Systems, Cary, NC, USA) (Figure 7).

## 3. Discussion

An appropriate future site for restoration was determined by performing diagnostic virtual tooth arrangements in the CAD software. These arrangements were reevaluated with respect to the relationship of the underlying alveolar bone in the implant planning software [15]. With regard to the immediate placement of provisional restorations, previous methods directly applied flowable composite resin onto the temporary abutment, or made impressions by using polyether or polyvinylsiloxane materials [29,30,31,32]. However, these methods may cause irritation to soft tissues and carry the risk of contamination because they involve manipulation around the surgical field.

In the present case, the immediate provisional restoration was produced without contact with conventional impression materials, which could minimize the risk of contamination. In addition, peri-implant soft tissues may have been less stimulated because the restoration was fabricated in a fully contoured form at a dental laboratory and did not require chairside relining.

Several techniques have been suggested to accurately transfer the peri-implant gingival tissue around the provisional restoration into the definitive cast [33,34,35,36,37,38,39]. In contrast to these physical methods, a digital approach was adopted in the present case. The intraoral impression was obtained first with the provisional restoration inserted state, followed by the trimming around the provisional restoration using a cutting tool in the software. Then, the peri-implant soft tissue impressions, with and without scan bodies, were added on to the previous digital intraoral impression. This procedure reduced the amount of time-dependent peri-implant tissue shrinkage [40]. The custom abutments and definitive restoration were placed without eliciting an uncomfortable feeling in the patient.

Considering the patient’s young age, future residual adult growth—which occurs very slowly over decades—may cause infraocclusion of the implant restoration [41,42,43]. Hence, the patient was informed of the need for maintenance adjustments and the potential requirement for a new prosthesis in the future. Chair time was reduced because of the comprehensive teamwork and the use of predesigned virtual teeth data.

The labial gingival contours clearly indicated reduced volume after extraction over the 1-year follow-up period in the present case, although efforts to rehabilitate the soft tissue contours were accompanied by implant placement. The most significant reduction in soft tissue contours occurred during the first 2 months postoperation. Controlled clinical studies are necessary to elucidate both short- and long-term changes of gingival contours after extraction and/or implantation.

## 4. Conclusions

The present report described the satisfactory rehabilitation of an anterior tooth region with multiple implants on which a provisional restoration was placed immediately after the surgery. The provisional restoration was easily fabricated by utilizing predesigned virtual teeth data in the CAD software. Dimensional changes around the sites of rehabilitation were visualized with reverse engineering software over a 1-year period, from the time of implant placement to the time 6 months after placement of the definitive restoration.

## Figures and Tables

**Table 1:** Time points at which digital intraoral impressions were made.

Digital Intraoral Impressions	First	Second	Third	Fourth	Fifth
Time points	Immediately after extraction	2 months after implant placement	4 months after implant placement	6 months after implant placement	1 year after implant placement
Treatments provided	Extraction, immediate implant placement, and immediate provisionalization	Exchange of provisional restoration	Final impression-making	Placement of definitive restoration	Routine follow-up

## Supplementary Materials

The following are available online at https://www.mdpi.com/2077-0383/8/4/428/s1, Video S1: Automatic fitting of the arbitrarily arranged virtual teeth onto the predesigned virtual teeth.

## References

[1] Van Noort R. (2012). The future of dental devices is digital. Dent. Mater..

[2] Block M.S. (2018). Dental implants: The last 100 years. J. Oral Maxillofac. Surg..

[3] Patel N. (2010). Integrating three-dimensional digital technologies for comprehensive implant dentistry. J. Am. Dent. Assoc..

[4] Zimmermann M., Mehl A., Mormann W.H., Reich S. (2015). Intraoral scanning systems—A current overview. Int. J. Comput. Dent..

[5] Marchack C.B., Chew L.K. (2015). The 10-year evolution of guided surgery. J. Calif. Dent. Assoc..

[6] Sarment D.P., Sukovic P., Clinthorne N. (2003). Accuracy of implant placement with a stereolithographic surgical guide. Int. J. Oral Maxillofac. Implant..

[7] Deeb G.R., Allen R.K., Hall V.P., Whitley D., Laskin D.M., Bencharit S. (2017). How accurate are implant surgical guides produced with desktop stereolithographic 3-dimentional printers?. J. Oral Maxillofac. Surg..

[8] Nickenig H.J., Wichmann M., Hamel J., Schlegel K.A., Eitner S. (2010). Evaluation of the difference in accuracy between implant placement by virtual planning data and surgical guide templates versus the conventional free-hand method-a combined in vivo-in vitro technique using cone-beam ct (part ii). J. Cranio Maxillofac. Surg..

[9] Rangel F.A., Maal T.J.J., de Koning M.J.J., Bronkhorst E.M., Berge S.J., Kuijpers-Jagtman A.M. (2018). Integration of digital dental casts in cone beam computed tomography scans-a clinical validation study. Clin. Oral Investig..

[10] Garber D.A., Belser U.C. (1995). Restoration-driven implant placement with restoration-generated site development. Compend. Contin. Educ. Dent..

[11] Pesun I.J., Gardner F.M. (1995). Fabrication of a guide for radiographic evaluation and surgical placement of implants. J. Prosthet. Dent..

[12] Almog D.M., Torrado E., Meitner S.W. (2001). Fabrication of imaging and surgical guides for dental implants. J. Prosthet. Dent..

[13] Kopp K.C., Koslow A.H., Abdo O.S. (2003). Predictable implant placement with a diagnostic/surgical template and advanced radiographic imaging. J. Prosthet. Dent..

[14] Ritter L., Neugebauer J., Dreiseidler T., Rothamel D., Cizek J., Karapetian V.E., Mischkowski R.A., Bindl A., Zoller J.E. (2009). 3D X-ray meets CAD/CAM dentistry: A novel procedure for virtual dental implant planning. Int. J. Comput. Dent..

[15] Lanis A., Alvarez Del Canto O. (2015). The combination of digital surface scanners and cone beam computed tomography technology for guided implant surgery using 3shape implant studio software: A case history report. Int. J. Prosthodont..

[16] Stimmelmayr M., Guth J.F., Erdelt K., Edelhoff D., Beuer F. (2012). Digital evaluation of the reproducibility of implant scanbody fit—An in vitro study. Clin. Oral Investig..

[17] Van Nimwegen W.G., Goene R.J., van Daelen A.C., Stellingsma K., Raghoebar G.M., Meijer H.J. (2016). Immediate implant placement and provisionalisation in the aesthetic zone. J. Oral Rehabil..

[18] De Bruyn H., Raes S., Ostman P.O., Cosyn J. (2014). Immediate loading in partially and completely edentulous jaws: A review of the literature with clinical guidelines. Periodontology.

[19] Levin B.P. (2011). Immediate temporization of immediate implants in the esthetic zone: Evaluating survival and bone maintenance. Compend. Contin. Educ. Dent..

[20] Bruno V., Badino M., Sacco R., Catapano S. (2012). The use of a prosthetic template to maintain the papilla in the esthetic zone for immediate implant placement by means of a radiographic procedure. J. Prosthet. Dent..

[21] Bruno V., O’Sullivan D., Badino M., Catapano S. (2014). Preserving soft tissue after placing implants in fresh extraction sockets in the maxillary esthetic zone and a prosthetic template for interim crown fabrication: A prospective study. J. Prosthet. Dent..

[22] Sohn D.S., Bae M.S., Heo J.U., Park J.S., Yea S.H., Romanos G.E. (2011). Retrospective multicenter analysis of immediate provisionalization using one-piece narrow-diameter (3.0-mm) implants. Int. J. Oral Maxillofac. Implant..

[23] Lauritano D., Grassi R., di Stasio D., Lucchese A., Petruzzi M. (2014). Successful mandible rehabilitation of lower incisors with one-piece implants. J. Med. Case Rep..

[24] Siadat H., Alikhasi M., Beyabanaki E. (2017). Interim prosthesis options for dental implants. J. Prosthodont..

[25] AlKudmani H., Al Jasser R., Andreana S. (2017). Is bone graft or guided bone regeneration needed when placing immediate dental implants? A systematic review. Implant. Dent..

[26] Hammerle C.H., Lang N.P. (2001). Single stage surgery combining transmucosal implant placement with guided bone regeneration and bioresorbable materials. Clin. Oral Implant. Res..

[27] Roccuzzo M., Gaudioso L., Bunino M., Dalmasso P. (2014). Long-term stability of soft tissues following alveolar ridge preservation: 10-year results of a prospective study around nonsubmerged implants. Int. J. Periodontics Restor. Dent..

[28] Ackermann K.L. (2009). Extraction site management using a natural bone mineral containing collagen: Rationale and retrospective case study. Int. J. Periodontics Restor. Dent..

[29] Levin B.P., Wilk B.L. (2013). Immediate provisionalization of immediate implants in the esthetic zone: A prospective case series evaluating implant survival, esthetics, and bone maintenance. Compend. Contin. Educ. Dent..

[30] Testori T., Galli F., Capelli M., Zuffetti F., Esposito M. (2007). Immediate nonocclusal versus early loading of dental implants in partially edentulous patients: 1-year results from a multicenter, randomized controlled clinical trial. Int. J. Oral Maxillofac. Implant..

[31] Schincaglia G.P., Marzola R., Giovanni G.F., Chiara C.S., Scotti R. (2008). Replacement of mandibular molars with single-unit restorations supported by wide-body implants: Immediate versus delayed loading. A randomized controlled study. Int. J. Oral Maxillofac. Implant..

[32] Cooper L.F., Raes F., Reside G.J., Garriga J.S., Tarrida L.G., Wiltfang J., Kern M., de Bruyn H. (2010). Comparison of radiographic and clinical outcomes following immediate provisionalization of single-tooth dental implants placed in healed alveolar ridges and extraction sockets. Int. J. Oral Maxillofac. Implant..

[33] Hinds K.F. (1997). Custom impression coping for an exact registration of the healed tissue in the esthetic implant restoration. Int. J. Periodontics Restor. Dent..

[34] Buskin R., Salinas T.J. (1998). Transferring emergence profile created from the provisional to the definitive restoration. Pract. Periodontics Aesthetic Dent..

[35] Stumpel L.J., Haechler W., Bedrossian E. (2000). Customized abutments to shape and transfer peri-implant soft-tissue contours. J. Calif. Dent. Assoc..

[36] Jansen C.E. (1995). Guided soft tissue healing in implant dentistry. J. Calif. Dent. Assoc..

[37] Chee W.W., Cho G.C., Ha S. (1997). Replicating soft tissue contours on working casts for implant restorations. J. Prosthodont..

[38] Neale D., Chee W.W. (1994). Development of implant soft tissue emergence profile: A technique. J. Prosthet. Dent..

[39] Elian N., Tabourian G., Jalbout Z.N., Classi A., Cho S.C., Froum S., Tarnow D.P. (2007). Accurate transfer of peri-implant soft tissue emergence profile from the provisional crown to the final prosthesis using an emergence profile cast. J. Esthet. Restor. Dent..

[40] Joda T. (2015). Time-dependent supraimplant mucosa changes: Short communication. Int. J. Oral Maxillofac. Implant..

[41] Zitzmann N.U., Arnold D., Ball J., Brusco D., Triaca A., Verna C. (2015). Treatment strategies for infraoccluded dental implants. J. Prosthet. Dent..

[42] Gotfredsen K., Carlsson G.E., Jokstad A., Arvidson Fyrberg K., Berge M., Bergendal B., Bergendal T., Ellingsen J.E., Gunne J., Hofgren M. (2008). Implants and/or teeth: Consensus statements and recommendations. J. Oral Rehabil..

[43] Oesterle L.J., Cronin R.J. (2000). Adult growth, aging, and the single-tooth implant. Int. J. Oral Maxillofac. Implant..

